# Evaluating Procedural Performance: A Composite Outcome for Atrial Septal Defect and Patent Ductus Arteriosus Closures

**DOI:** 10.1016/j.jscai.2024.102459

**Published:** 2025-01-09

**Authors:** Oliver M. Barry, Babar S. Hasan, Nadeem Aslam, Sarosh P. Batlivala, Matthew A. Crystal, Sara M. Trucco, Todd Gudausky, Ralf J. Holzer, Jacqueline Kreutzer, George Nicholson, Michael L. O’Byrne, Brian P. Quinn, Surendranath R. Veeram Reddy, Arash Salavitabar, Brian A. Boe

**Affiliations:** a Division of Pediatric Cardiology, NewYork-Presbyterian – Morgan Stanley Children’s Hospital, Columbia University Medical Center, New York, New York; b Division of Cardiothoracic Sciences, Sindh Institute of Urology and Transplantation, Karachi, Pakistan; c The Heart Institute, Cincinnati Children’s Hospital and Department of Pediatrics, University of Cincinnati College of Medicine, Cincinnati, Ohio; d Heart & Vascular Institute, UPMC Children’s Hospital of Pittsburgh, Pittsburgh, Pennsylvania; e Division of Pediatric Cardiology, Children’s Wisconsin, Medical College of Wisconsin, Milwaukee, Wisconsin; f Department of Pediatrics, UC Davis Medical Center, UC Davis Children’s Hospital, Sacramento, California; g Division of Pediatric Cardiology, Monroe Carell Jr. Children’s Hospital at Vanderbilt University Medical Center, Nashville, Tennessee; h Division of Cardiology, The Children’s Hospital of Philadelphia and Department of Pediatrics Perelman School of Medicine at The University of Pennsylvania, Philadelphia, Pennsylvania; i Department of Cardiology, Boston Children’s Hospital and Department of Pediatrics, Harvard Medical School, Boston, Massachusetts; j Department of Pediatrics, UT Southwestern Medical Center, Dallas, Texas; k The Heart Center, Nationwide Children’s Hospital and Department of Pediatrics, The Ohio State University College of Medicine, Columbus, Ohio; l Department of Cardiology, Joe DiMaggio Children’s Hospital, Hollywood, Florida

**Keywords:** atrial septal defect closure, patient ductus arteriosus occlusion, procedural performance

## Abstract

**Background:**

Technical success (TS) and procedural safety (PS) have been reported individually for transcatheter atrial septal defect (ASD) and patent ductus arteriosus (PDA) closures. A composite procedural performance (PP) metric as a patient-centered strategy has not been developed or studied.

**Methods:**

A multicenter expert working group created PP metrics for ASD and PDA device closures as a composite of TS and PS. TS criteria were defined and categorized into 3 classes (optimal, satisfactory, and unsatisfactory). PS was defined using established adverse event (AE) definitions from the Congenital Cardiac Catheterization Project on Outcomes (C3PO) registry. PP was divided into 3 outcome classes (I to III). Retrospective C3PO data were collected for all cases of isolated ASD and PDA closure from 2014 through 2017. Exclusion criteria included complex congenital heart disease, significant comorbidities, ASD patients with multiple defects or ≥2 deficient rims, and PDA patients weighing <6 kg or with pulmonary hypertension. Factors correlating with class III (suboptimal) PP were analyzed.

**Results:**

A total of 542 ASD and 688 PDA closure cases were included. Most ASD cases (99%) had optimal or satisfactory TS while 1% had a high severity AE. Class III PP occurred in 2% of ASD cases, mostly due to new mitral valve insufficiency. There were no identified patient or procedural factors associated with class III PP for ASD closures. Optimal or satisfactory TS occurred in 98% of PDA cases, with high severity AEs in <1%. Class III PP occurred in 2% of PDA cases, predominantly due to new arch obstruction, and was associated with younger age (*P* < .001) and lower weight (*P* = .001).

**Conclusions:**

This study introduces PP as a composite variable to comprehensively measure outcomes of standard-risk ASD and PDA device closure. The incorporation of both TS and PS aims to better reflect patient outcomes compared to individual measurements alone. PP may serve as a valuable tool for identifying areas for further investigation and quality improvement.

## Introduction

Research on procedures for congenital cardiac catheterization has traditionally focused on assessing either technical success (TS) or procedural safety (PS).^1^, ^2^, ^3^ This type of outcome research has played a crucial role in enhancing the safety and efficacy of transcatheter procedures, although it has assessed procedural characteristics within a relatively narrow scope. The development of a procedural performance (PP) metric combining both TS and PS has the potential to provide a useful tool to further optimize transcatheter procedures and improve outcomes of congenital cardiac surgery.^4^, ^5^, ^6^, ^7^, ^8^

The International Quality Improvement Collaborative Congenital Heart Disease Catheterization Registry (IQIC-CHDCR) recently introduced an innovative composite variable for 5 relatively frequently performed congenital interventions such as aortic valvuloplasty, atrial septal defect (ASD) closure, vascular rehabilitation of coarctation, patent ductus arteriosus (PDA) closure, and pulmonary valvuloplasty. For device closure procedures, this variable combines TS descriptors (eg, residual shunt), adverse events (AEs), and ultimate disposition (ie, elective home discharge).^9^ While the concept of this metric has been introduced in few centers, it requires further refinement of its variable components using a large data set.

The aim of this project was to evaluate the composite PP metric introduced by the IQIC-CHDCR, specifically for patients undergoing standard-risk transcatheter closures of ASD and PDA, utilizing the larger multicenter Congenital Cardiac Catheterization Project Outcome (C3PO) data set (https://c3po-r3.chboston.org/#/home).^10^ A prior study evaluated a PP metric for aortic and pulmonary balloon valvuloplasty.^11^ In addition, the study aimed to identify patient and procedural characteristics associated with optimal PP.

## Methods

### Variable development

To formulate a composite variable for PP, it was necessary to establish criteria for both TS and PS. The development of this innovative outcome variable was built upon the foundation of a previously published composite variable from IQIC-CHDCR.^9^ The published criteria were revised by an expert panel comprising physicians from various C3PO sites who served as a working group (WG). Relevant technical metrics and short- and long-term patient outcomes from earlier publications are summarized in Supplemental Table S1.^12^, ^13^, ^14^, ^15^, ^16^ Final PP criteria were selected based on insights from this prior outcome research and the expert opinions of the WG; criteria deemed acceptable to >80% of the WG were incorporated.

Criteria for TS were established by considering factors such as residual shunting and the development of anatomic/additional/cardiac lesions associated with device placement. TS for each case was categorized as optimal, satisfactory, or unsatisfactory (Table 1). In the case of ASD devices, residual shunting across the defect was classified as none, mild to moderate (flow <3 mm in diameter), or severe (flow ≥3 mm in diameter) as determined on postprocedural transthoracic echocardiography and using residual shunt sizes based on previous studies (Supplemental Table S1).^12^^,^^13^ Mitral regurgitation (MR) was captured in the C3PO registry as a binary variable of “new or changed mitral valve insufficiency.” Post-device MR severity was self-reported by centers, but pre-device MR information was not available. Based on expert consensus and clinical practice, if the new/changed MR was reported as trivial, this was a criterion for a satisfactory TS. If the post-device MR was mild or worse, this was a criterion for an unsatisfactory TS. Therefore, an optimal TS for ASD closure could only be achieved if the patient left the catheterization laboratory with no significant residual shunt and no new MR. Residual shunting after PDA device closure was categorized as none, mild residual shunting not necessitating intervention (either catheterization or operation), or greater than or equal to moderate residual shunting requiring intervention. The emergence of new lesions after PDA device closure included stenosis of the left pulmonary artery and/or aorta. The peak gradient (PG) measured by catheterization and/or peak velocity (PV) measured by echocardiography were used to classify TS (optimal: PG <16 mm Hg or PV <2 m/s; satisfactory: PG 16 to 36 mm Hg or PV 2 to 3 m/s; unsatisfactory: PG >36 mm Hg or PV >3 m/s). The availability of these gradients relied upon self-reporting by participating centers.

**Table 1:** Patient characteristics and outcomes by device closure cohort.

	ASD device closure (n = 542)	PDA device closure (n = 688)
Male sex	181 (34%)	224 (33%)
Age at catheterization, y	6.9 (4.5, 13.9)	3.0 (1.3, 5.5)
Weight at catheterization, kg	23.2 (16.6, 51.2)	13.8 (10.0, 19.6)
Technical success	n = 528	n = 541
Optimal	435 (80%)	513 (95%)
Satisfactory	102 (19%)	16 (3%)
Unsatisfactory	5 (1%)	12 (2%)
Length of stay, d	1 (1, 1) (n = 480)	0 (0, 1) (n = 585)
Unplanned catheterization or surgery	2 (<1%)	1 (<1%)
Any level 4/5 adverse event	3 (1%)	1 (<1%)
Death within 72 h	0 (0%)	0 (0%)
Procedural performance	n = 528	n = 541
Class I	403 (76%)	478 (88%)
Class II	117 (22%)	51 (10%)
Class III	8 (2%)	12 (2%)

Values are n (%) or median (25th, 75th percentile).

ASD, atrial septal defect; PDA, patent ductus arteriosus.

PS was determined utilizing the AEs reported by the participating sites, detailing severity levels and categorization as previously outlined.^17^, ^18^, ^19^ AEs with severity levels 3 to 5 were deemed severe AEs and those at severity levels 4 and 5 were labeled as high severity adverse events (HSAEs). The categorization and severity scoring of AEs were independently reviewed for accuracy and consistency as part of the standard audit process conducted by C3PO.

By amalgamating the individual TS and PS for each procedure, PP was categorized into 3 classes (I to III), as outlined in Table 1. An outcome with optimal TS and AE severity ranging from 0 to 2 was classified as a class I PP (ideal). An unsatisfactory TS and/or AE severity of 4 to 5 were categorized as a class III PP (suboptimal) outcome. PP classification was determined based on the worst criteria variable. In other words, for a case to be classified as class I, all criteria (TS and PS) must be met, while any single criteria falling within unsatisfactory TS or HSAEs would categorize the case as suboptimal (class III).

### Case selection and data collection

A multicenter analysis was conducted using data from the C3PO registry, encompassing all cases of standard-risk ASD and PDA device closures recorded from 2014 to 2017. Exclusions were made for patients with complex congenital heart disease and significant comorbidities such as genetic syndromes, chronic lung disease, and renal insufficiency. Procedure-specific exclusion criteria for the ASD cohort included >1 ASD and having ≥2 deficient rims. In the PDA cohort, exclusions were made for cases with weight <6 kg and pulmonary hypertension (defined as systolic pulmonary arterial pressure greater than systolic systemic pressure). The 6 kg weight cutoff for PDA device closure was chosen to align with the recommendations in the instructions for use documentation for US Food and Drug Administration-approved PDA devices during the study period.^20^ Cases were excluded from the final analysis if there were missing data related to PP.

Data entered into the C3PO registry are self-reported and audited annually. Collected patient data for the study included age and weight at the time of catheterization. For ASD device closure, procedural data included presence and location of deficient rims. Procedural data for PDA device closure included information about PDA shape, PDA size, and the delivery technique. Comprehensive AE details were collected for all cases. Outcome variables examined included length of stay (LOS) and occurrences of unplanned or emergent surgery or death within 72 hours of the cardiac catheterization procedure.

### Statistical analysis

Patient characteristics and outcomes were summarized separately for subjects in the ASD and PDA device closure cohorts. Categorical variables are presented as frequencies and percentages, while continuous variables are expressed as medians with IQRs denoting the 25th and 75th percentiles. To explore factors associated with suboptimal efficacy within each cohort, patients with class III PP were compared to those with classes I and II combined using Fisher exact test or the Wilcoxon rank sum test. The analyses were conducted using Stata version 16 (Stata Corp).

Approval for this study was obtained from the Boston Children’s Hospital Institutional Review Board, and it was supported by data-sharing agreements between Boston Children’s Hospital and the C3PO participating sites as required.

## Results

**Figure 1:**
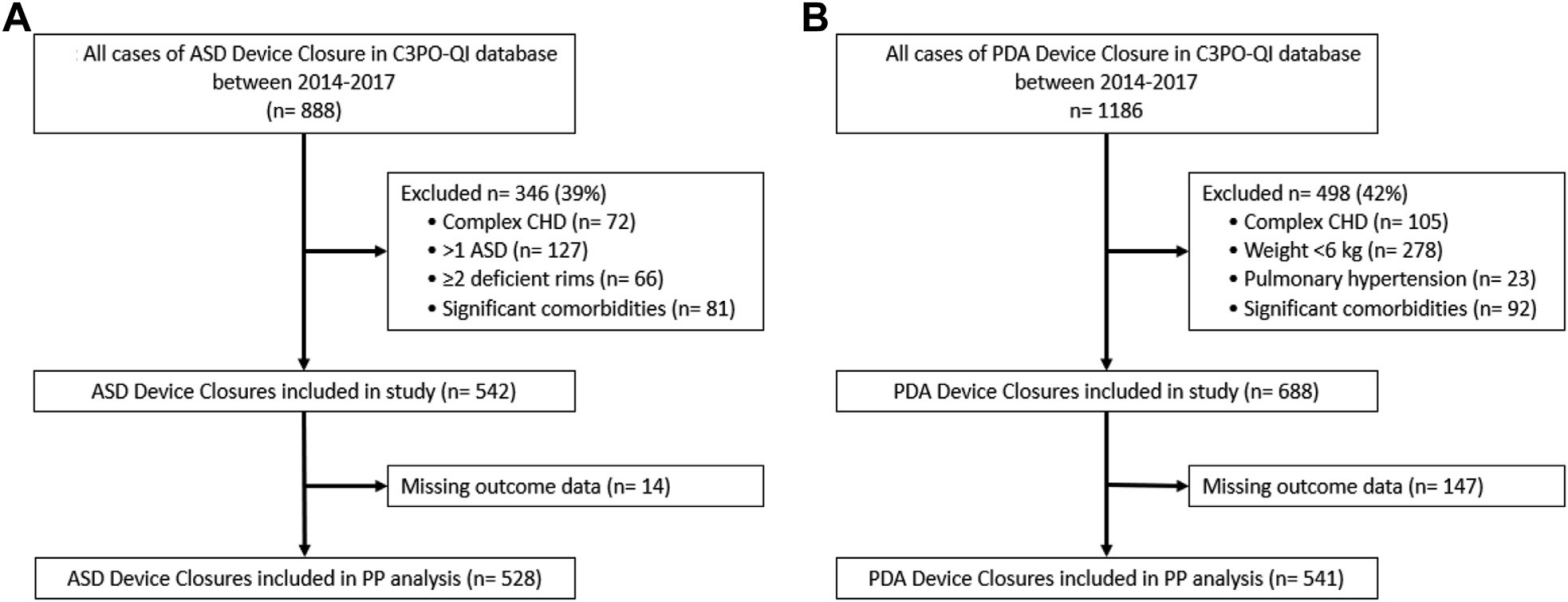
**Flowchart of study patients.** PP class (I, II, and III) and y-axis is the percentage of patients in that particular PP class. ASD, atrial septal defect; C3PO-QI, Congenital Cardiac Catheterization Project on Outcomes Quality Improvement; CHD, congenital heart disease; PDA, patent ductus arteriosus; PP, procedural performance.

During the study period, the C3PO registry documented 888 isolated ASD device closures and 1186 isolated PDA device closures. A total of 346 (39%) ASD cases were excluded due to the presence of >1 ASD, significant comorbidities, complex congenital heart disease, and ≥2 deficient rims. Similarly, 498 (42%) PDA cases were excluded based on weight <6 kg, complex congenital heart disease, significant comorbidities, and/or pulmonary hypertension. As a result, 542 ASD device closures and 688 PDA device closures were studied (Figure 1). Notably, outcome data related to PP were missing for 14 ASD device closure patients and 147 PDA device closure patients, resulting in the evaluation of PP in 528 ASD device closures and 541 PDA device closures. Detailed information for each procedure is provided below.

### ASD closures

Patients undergoing ASD device closure had a median age of 6.9 years (IQR, 4.5-13.9) and a weight of 23.2 kg (IQR, 16.6-51.2) at the time of the procedure (Table 1). Optimal and satisfactory TS was observed in 99% of cases (n = 537), with only 3 patients (1%) experiencing a HSAE. The median LOS after ASD device closure was 1 day.

**Figure 2:**
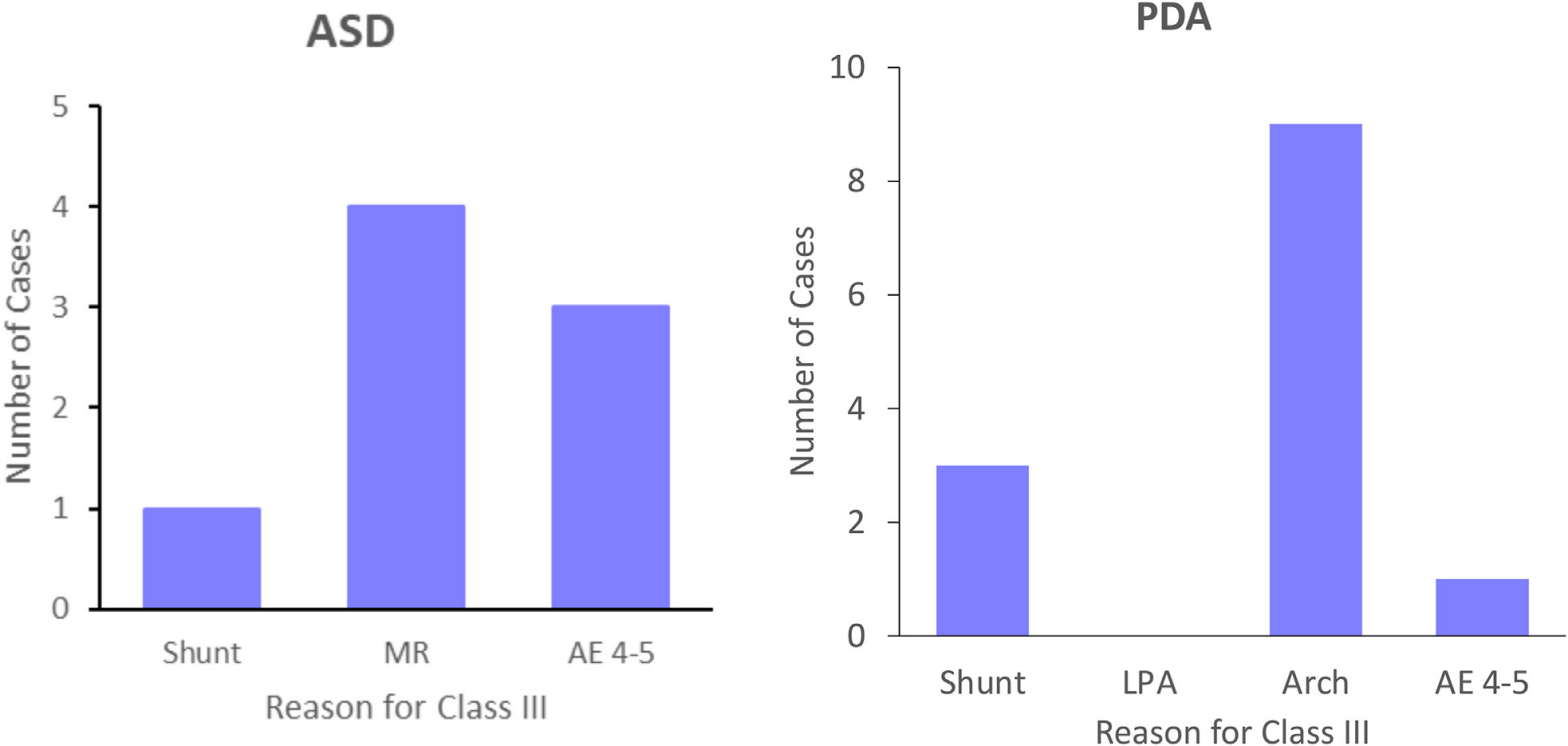
**Reason for class III procedural performance in ASD and PDA device closure.** The graphs show the number of patients for each reason for class III procedural performance. AE, adverse event; Arch, aortic arch stenosis; ASD, atrial septal defect; LPA, left pulmonary artery stenosis; MR, mitral valve regurgitation.

The majority of patients achieved class I and II PP (n = 520, 98%). Class III PP was primarily attributed to unsatisfactory TS due to mild or moderate MR (n = 4), AE noted (n = 3), and severe residual shunting (n = 1) (Figure 2). The one patient with moderate MR had a deficient retroaortic rim. There were 3 level 4 AEs, including a device embolization leading to worsening MR, a device embolization to the left ventricular outflow tract requiring surgical removal, and a hemothorax related to transhepatic access (Supplemental Table S2). There were no factors associated with class III PP ASD device closure (Table 2).

**Table 2:** Factors and class III procedural performance – ASD device closure.

	Class I/II (n = 520)	Class III (n = 8)	*P*
Male sex	175/514 (34%)	3/8 (38%)	1.0
Age at catheterization, y	6.8 (4.5, 14.0)	11.0 (6.3, 16.6)	.32
Weight at catheterization, kg	23.1 (16.6, 51.1) (n = 518)	24.5 (18.3, 51.6) (n = 8)	.97
One deficient rim	56 (11%)	2 (25%)	.22

Values are n (%) or median (25th, 75th percentile).

Comparisons were made using Fisher exact test or the Wilcoxon rank sum test.

ASD, atrial septal defect.

### PDA closures

Patients undergoing PDA device closure had a median age of 3 years (IQR, 1.3-5.5) and a weight of 13.8 kg (IQR, 10-19.6) at the time of the procedure (Table 1). Optimal and satisfactory TS was achieved in 98% of cases (n = 529), with only 1 patient (<1%) experiencing a HSAE. The majority of patients were discharged home later the same day after PDA device closure (Supplemental Table S3).

Class I and II PP was accomplished in the majority of patients (n = 529, 98%). Class III PP was primarily because of unsatisfactory TS due to arch obstruction (n = 9) and greater than or equal to moderate shunting through or around the device (n = 3) (Figure 2). A single level 4 AE involved an embolized PDA device requiring surgical removal. Class III PP PDA device closures involved younger (0.9 vs 3.1 years; *P* < .001) and smaller (9.1 vs 14 kg; *P* = .001) patients compared to class I and II cases (Table 3).

**Table 3:** Factors and class III procedural performance – PDA device closure.

	Class I/II (n = 529)	Class III (n = 12)	*P*
Male sex	170 (33%)	4 (36%)	.76
Age at catheterization, y	3.1 (1.4, 5.5)	0.9 (0.8, 1.6)	<.001
Weight at catheterization, kg	14.0 (10.1, 19.8)	9.1 (7.9, 9.7)	.001
PDA minimal diameter, mm	2.0 (1.9, 3.0)	2.6 (2.0, 3.0)	.55
PDA length, mm	9 (7, 12)	8 (7, 13)	.84
PDA classification			
Conical (A)	380 (73%)	6 (50%)	.077
Window (B)	6 (1%)	1 (8%)	
Tubular (C)	56 (11%)	2 (17%)	
Complex (D)	23 (4%)	1 (8%)	
Elongated (E)	58 (11%)	2 (17%)	
Delivery technique			
Prograde	353 (78%)	9 (75%)	.73
Retrograde	97 (22%)	3 (25%)	

Values are n (%) or median (25th, 75th percentile).

PDA, patent ductus arteriosus.

## Discussion

The PP composite outcome variable was developed by combining previously published data and the expertise of our expert WG. The application of this composite variable demonstrated excellent results for standard-risk transcatheter ASD device closure and PDA device closure procedures. The occurrence of suboptimal, class III PP (2%) was primarily related to the presence of MR in ASD device closures and arch obstruction in PDA device closures (Central Illustration). Although none of the factors explored were found to have a statistically significant association with class III PP in ASD closures, class III PDA closures were associated to younger and smaller patients.

**Central Illustration:**
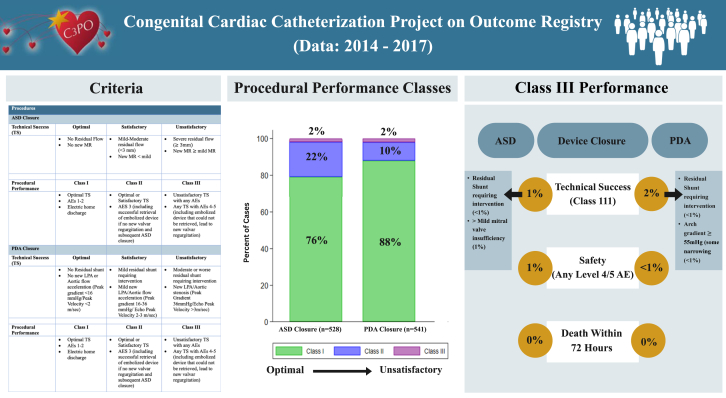
**Congenital Cardiac Catheterization Project on Outcome Registry.** The table on the left outlines the criteria developed for each procedure to categorize technical success (TS) and procedural performance (PP). The central bar graph depicts the PP classes of the cases evaluated in this study and highlights the low percentage of cases with a class III PP outcome. The reasons for class III PP are highlighted in the chart on the right. Class III outcomes for both procedures were rare. For ASD closure cases, the reasons for class III PP were due to both TS and safety. For PDA closure cases, unsatisfactory TS was the reason for class III PP. AE, adverse event; ASD, atrial septal defect; MR, mitral valve regurgitation; LPA, left pulmonary artery; PDA, patent ductus arteriosus.

This study aimed to create and assess PP for transcatheter ASD and PDA device closures to better quantify procedural outcomes in standard-risk cases to limit confounding variables and illustrate the face validity of the variable. The combination of high TS and PS (low incidence of AEs) of these procedures in this cohort were similar to other published cohorts, predominantly showing class I and II PP outcomes.^9^^,^^12^^,^^14^^,^^15^^,^^21^ The short hospital LOS and the absence of unexpected surgeries reflects the standard- or low-risk patient cohort in this study. In its current form, PP for simple ASD and PDA device closures will provide a small opportunity for improvement due to the high rate of optimal performance, which limits the metric’s applicability as a quality improvement tool. An ideal PP metric must be applicable across most procedural risk levels, in addition to including a multitude of patient and procedural variables. ASD device closure in a healthy pediatric patient differs significantly from that in an adult patient with pulmonary hypertension and/or heart failure. Future development of PP for complex procedures and higher-risk patient populations (ie, fenestrated ASD closures, ASD closures with deficient rims, premature PDA closures) will enhance its applicability. Using the PP metric in these cases and patient cohorts with presumed lower rates of optimal performance will provide more opportunities for quality improvement. As an example, looking at the PP for device closure of ASDs with deficient rims may give us a better understanding of the optimal device to use. Is there a device that provides improved TS? Is improved TS achieved at the expense of PS? Evaluation of the PP in this setting would allow a more complete comparison of types of devices, which could guide operators toward the best device for a particular lesion and/or to refer the patient for surgical repair if the optimal PP was not achieved with any device. However, there are rare instances of high-risk/salvage procedures that need to be acknowledged, and potentially excluded, when utilizing PP as a quality improvement tool.

We found the causes of class III PP are potentially quite variable. Therefore, rather than simply identifying the outcome as suboptimal, the PP variable allows us to understand the major categories to address to improve future outcomes. In standard-risk ASD closures, there were no identified factors associated with class III PP. Approximately 1% of patients undergoing ASD device closure had mild (n = 3) or moderate (n = 1) MR, leading to the majority of cases having class III PP. Although this limited retrospective data set could not attribute the MR to the ASD device closure, the more pressing question is the long-term impact of the MR. In a study of 227 adult and pediatric patients undergoing ASD device closure, there was no significant change in the degree of MR with the procedure, although 33 patients had a change in MR (20 progressed and 13 improved) in the 1.2 years of follow-up.^22^ Further development of PP in ASD device closure will likely require evaluation of the change in MR from the procedure, change in MR over time, and any medical interventions due to the MR.

Suboptimal (class III) PP in patients undergoing PDA device closure was associated with younger and smaller patients. This finding may be due to the devices utilized during the study period (2014-2017) with larger aortic ends that can lead to obstruction, which has been previously described in rare cases.^23^, ^24^, ^25^ Currently available devices designed for deployment completely within the ductus arteriosus may lead to improved PP. Although these current devices are primarily used in neonates, who were excluded from this study, further evaluation in premature PDA occlusion may lead to further opportunities for procedural improvement.

The challenges related to transcatheter device placement metrics include adapting to device modifications and development along with changes in procedural technique. Future studies need to account for developments such as bioabsorbable ASD devices, newer PDA occlusion devices, and evolving techniques for PDA occlusion, especially in the premature infant population, as noted above. Though our study provides insights into the immediate outcomes for current devices, future work should correlate PP with long-term follow-up data, including freedom from reintervention, morbidity, mortality, and patient-reported outcomes.

There are many limitations of the study that need to be acknowledged. The lack of robust published evidence supporting our composite metric of PP required expert consensus from a WG. Additionally, there are significant limitations of the available data captured by the C3PO registry, including clinically important details about the degree of MR after ASD closure, size of residual shunts, and echocardiographic data regarding left pulmonary artery stenosis and aortic obstruction after PDA device placement. The clinical significance of residual shunts, such as a 3-mm residual shunt after ASD closure, is also unclear and must be acknowledged. Evaluation of the metric was performed utilizing a limited retrospective data set. C3PO recently published a new stratification of AE severity, which may improve the PP metric.^26^ The analysis was performed in standard-risk cases, which limits the generalizability of our study, and our results cannot be extrapolated to more complex defects and patients. Future work involves validating PP on more complex cohorts and assessing its impact on short- and long-term patient outcomes in a prospective manner. Incorporation of additional patient and procedural factors (such as genetic disorders, device type/size, anatomic variants, etc) may provide better understanding of the factors associated with PP for a larger cohort of patients.

## Conclusions

PP metrics are designed to offer a comprehensive outcome that encompasses both TS and PS. Notably, standard-risk ASD and PDA closure procedures have showcased high PP. The integration of both TS and PS into a composite metric holds the potential to portray patient outcomes more accurately, surpassing the isolated measurement of each outcome. This holistic approach aids in identifying specific areas that warrant further investigation and quality improvement initiatives.
