# Supplementary material for: Evaluating Procedural Performance: A Composite Outcome for Atrial Septal Defect and Patent Ductus Arteriosus Closures
Source: J Soc Cardiovasc Angiogr Interv. 2025 Jan 9;4(2):102459. doi: 10.1016/j.jscai.2024.102459 (PMC11916790; doi:10.1016/j.jscai.2024.102459)
Supplement: Supplementary Table S1 [file mmc1.docx]

**Supplementary Table S1: Evidence Base for Technical Success Criteria**

| Evidence Base for Technical Success Criteria | |
| --- | --- |
| *Source* | *Relevant Findings* |
|  |  |
| ASD Device Closure | |
| Everett et al., *Pediatr Cardiol*. 2009 Apr | - Technical success defined as device placement in defect at end of case |
|  | - Technical outcomes evaluated for residual shunting, defined as none/trivial (<1mm), small (1-2mm) and moderate (>2mm) |
| Du et al., *JACC.* 2002 June | Comparison of outcomes between surgical and transcatheter ASD closure |
|  | - Successful ASD closure defined as:   - Small (1-2mm) or less residual shunting |
|  |  |
| PDA Device Closure |  |
| Moore et al., *JACC: Cardiovasc Inter.* 2014 Dec | Results of Pivotal Study and Continued Access Study of the Nit-Occlud PDA Device   - Technical success defined as device in place at end of procedure |
| El-Said et al., *JAHA*. 2013 Oct | - Technical success defined as the patient leaving the catheterization laboratory with a coil or device in the PDA with no more than a tiny residual shunt based on angiography |
| Magee et al., *Eur Heart J.* 2001 Oct | - Suboptimal outcomes were defined with various elements including:   - Need for a further procedure due to residual flow   - Persistent hemolysis   - Significant flow disturbance in adjacent structures using a Doppler velocity of > 2 m/s |
