# Supplementary material for: Evaluating Procedural Performance: A Composite Outcome for Atrial Septal Defect and Patent Ductus Arteriosus Closures
Source: J Soc Cardiovasc Angiogr Interv. 2025 Jan 9;4(2):102459. doi: 10.1016/j.jscai.2024.102459 (PMC11916790; doi:10.1016/j.jscai.2024.102459)
Supplement: Supplementary Table S2 [file mmc2.docx]

**Supplemental Table S2: Outcomes for ASD Device Closure**

| **Outcome** | N (%) |
| --- | --- |
| **Residual shunt size** |  |
| None | 478 (88%) |
| Minimal | 55 (10%) |
| Moderate | 2 (<1%) |
| Severe | 1 (<1%) |
| Missing | 6 (1%) |
| **>Mild mitral valve insufficiency (post-procedure)** |  |
| Yes | 4 (1%) |
| No | 528 (97%) |
| Missing | 10 (2%) |
| **Highest severity adverse event** |  |
| None | 505 (93%) |
| 1 | 1 (<1%) |
| 2 | 18 (3%) |
| 3 | 15 (3%) |
| 4 | 3 (1%) |
| 5 | 0 (0%) |
| **Elective home discharge†** |  |
| Yes | 463 (85%) |
| No | 79 (15%) |

† Discharge destination home or time to discharge 1 day after cath procedure
