# Supplementary material for: Evaluating Procedural Performance: A Composite Outcome for Atrial Septal Defect and Patent Ductus Arteriosus Closures
Source: J Soc Cardiovasc Angiogr Interv. 2025 Jan 9;4(2):102459. doi: 10.1016/j.jscai.2024.102459 (PMC11916790; doi:10.1016/j.jscai.2024.102459)
Supplement: Supplementary Table S3 [file mmc3.docx]

**Supplemental Table S3: Outcomes for PDA Device Closure**

| **Outcome** | N (%) |
| --- | --- |
| **Residual shunt** |  |
| None or trivial flow through device | 626 (91%) |
| Shunt around device (mild/small) | 17 (2%) |
| Shunt around device (moderate or more) | 3 (<1%) |
| Not assessed | 6 (1%) |
| Missing | 36 (5%) |
| **LPA obstruction (post-procedure)** |  |
| None | 553 (80%) |
| Not assessed | 89 (13%) |
| Missing | 46 (7%) |
| **Arch obstruction (post-procedure)** |  |
| None | 603 (88%) |
| Yes – angiographically (gradient not assessed) | 2 (<1%) |
| Yes – gradient ≥5 mmHg (some narrowing) | 1 (<1%) |
| Yes – gradient ≥5 mmHg (no narrowing) | 6 (1%) |
| Not assessed | 31 (5%) |
| Missing | 45 (7%) |
| **Highest severity adverse event** |  |
| None | 643 (93%) |
| 1 | 0 (0%) |
| 2 | 30 (4%) |
| 3 | 14 (2%) |
| 4 | 1 (<1%) |
| 5 | 0 (0%) |
| **Elective home discharge†** |  |
| Yes | 656 (95%) |
| No | 26 (4%) |
| Missing | 6 (1%) |

† Discharge destination home or time to discharge 1 day after cath procedure
